# Multi-Omics Pan-Cancer Profiling of *HSD17B10* Unveils Its Prognostic Potential, Metabolic Regulation, and Immune Microenvironment Interactions

**DOI:** 10.3390/biology14050567

**Published:** 2025-05-19

**Authors:** Tao Qi, Xiao Chang, Yiming Wang

**Affiliations:** School of Statistics and Applied Mathematics, Anhui University of Finance and Economics, Bengbu 233010, China; 3202200268@aufe.edu.cn

**Keywords:** *HSD17B10*, pan-cancer analysis, mitochondrial metabolism, immune microenvironment

## Abstract

This study systematically analyzed the expression and clinical significance of Hydroxysteroid 17-beta dehydrogenase type 10 (*HSD17B10*) in 33 cancers by integrating TCGA, GTEx, and other multi-omics databases. *HSD17B10* was highly expressed in 14 cancers, like GBM and LGG, but low in 5, such as KIRC. Its expression correlated closely with overall survival (OS) and disease-free survival (DFS). In GBM-LGG, LGG, and other cancers, high *HSD17B10* expression was linked to lower survival rates, indicating that it could be an independent prognostic marker. *HSD17B10* also had a two-way relationship with the tumor’s immune microenvironment. In cancers such as GBM-LGG, high expression correlated positively with immune/stromal scores. However, in most cancers like LUAD, it was negatively associated with B- and T-cell infiltration. Epigenetic analysis showed that low methylation in the *HSD17B10* promoter region might drive its high expression in tumors such as SARC, and specific methylation sites (e.g., CG26323797) were significantly related to patient survival. Functional enrichment analysis revealed that *HSD17B10* participated in tumor progression by regulating oxidative phosphorylation, mitochondrial metabolism, and RNA methylation. Single-cell and spatial transcriptome data further demonstrated that *HSD17B10* had a cell-type-specific expression pattern in colorectal cancer. This study provides a theoretical basis for *HSD17B10* as a pan-cancer prognostic marker and therapeutic target.

## Simple Summary

This study integrated multi-omics data from The Cancer Genome Atlas (TCGA) and Genotype-Tissue Expression (GTEx) databases. We found that Hydroxysteroid 17-beta dehydrogenase type 10 ( *HSD17B10*) exhibited distinct expression patterns in 33 types of cancers. It was highly expressed in 14 types of cancers, including glioblastoma multiforme (GBM) and low-grade glioma (LGG), but was lowly expressed in five types of cancers, such as kidney renal clear cell carcinoma (KIRC). We conclude that its expression level is closely associated with patient survival rates, with high expression typically indicating a poorer prognosis. Moreover, *HSD17B10* not only has a complex relationship with the tumor immune microenvironment, but also participates in the mitochondrial energy metabolism of tumor cells. Single-cell and spatial transcriptome data further demonstrated that *HSD17B10* has a cell-type-specific expression pattern in colorectal cancer. It can be concluded that *HSD17B10* can serve as a reliable biomarker for cancer prognosis and a promising target for new cancer treatments. It can assist doctors in making more informed treatment decisions, improving treatment effectiveness, and enhancing patients’ quality of life.

## 1. Introduction

Cancer poses a serious public health challenge globally, significantly affecting the lives of millions of families around the world and imposing a heavy burden on the healthcare system [1]. Cancer is a malignant disease caused by uncontrolled cell growth. Its pathogenesis lies in normal cells undergoing gene mutations or other genetic alterations under the combined action of internal and external factors. They then lose the precise control ability of normal growth and differentiation [2]. These abnormal cells possess gene mutations, genomic instability, uncontrolled cell proliferation, abnormal differentiation, and enhanced invasion and metastasis capabilities [3]. Gene mutation, one of the main driving forces for tumor formation, can occur spontaneously, such as DNA replication errors, or be induced by external factors. It also significantly influences the clinical outcomes of cancer patients receiving molecular-targeted therapy and immunotherapy. Therefore, in-depth exploration of the functions and mechanisms of cancer-related genes is crucial for developing new treatment strategies and improving patient prognosis.

The *HSD17B10* gene encodes a multifunctional mitochondrial protein indispensable in various biological functions. This gene is located on chromosome Xp11.22, and its expression levels vary remarkably between different cancers [4]. Many studies have shown that this expression difference is closely related to tumors’ biological characteristics and patients’ survival rate. For example, in certain cancers, high expression of *HSD17B10* is associated with tumor invasiveness and drug resistance, while low expression may indicate a better prognosis [5]. Furthermore, SIRT3-regulated acetylation of *HSD17B10* influences cell growth and resistance to oxidative/starvation stress, which is essential in cancer treatment. By clarifying the expression patterns of *HSD17B10* in different cancers, targeted treatment plans are expected to be designed to inhibit its function in cancer cells, thus effectively suppressing tumor growth. Recent studies, through the analysis of the coexpression network of *HSD17B10* in bladder cancer (BLCA) and the analysis of the single-cell and spatial transcriptome of colorectal cancer (CRC), have further revealed the potential role of this gene in tumorigenesis and development [6]. However, the complete mechanism of *HSD17B10* in multiple cancers remains poorly understood.

In this study, we conducted a comprehensive pan-cancer analysis based on multiple databases and analytical methods. We systematically explored the expression characteristics and molecular mechanisms of *HSD17B10* across 33 cancer types and their relationship with clinical outcomes. We further classified cancer patients according to *HSD17B10*-related features. We verified significant differences in prognosis, gene mutations, tumor microenvironment, and drug sensitivity among different groups, providing new insights for cancer treatment and prognostic evaluation.

## 2. Methods

### 2.1. Data Sources

This research obtained gene expression data from The Cancer Genome Atlas (TCGA) and Genotype-Tissue Expression (GTEx) databases. This study obtained gene expression data from the TCGA and GTEx databases. The TCGA dataset covers 33 types of cancers and contains 10,967 tumor samples and 1219 standard samples; These standard samples serve as controls for the tumor samples. The GTEx dataset, which comprises 11,688 samples, provides the gene expression data of normal tissues for comparison with the tumor sample data in the TCGA database. Through this comparative analysis, the differences in gene expression between tumor tissues and normal tissues can be revealed more clearly. After obtaining data from the TCGA and GTEx databases, we removed any samples with missing values or low-quality measurements, which could potentially introduce biases into the analysis. We extracted the expression data of the *HSD17B10* gene based on gene annotation. Then, we used log2 transformation to standardize the data. This transformation can make the data more normally distributed, reduce heterogeneity and the impact of extreme values, and eliminate biases between different datasets. Next, we used the limma package 3.46.0 in R. for differential expression analysis and set the false discovery rate (FDR) < 0.05 and |log2 (fold change)| > 1 as thresholds. These thresholds can filter out false positives and ensure the reliability of the results.

### 2.2. HSD17B10 Gene Expression Analysis

Human Protein Atlas (HPA) was used to obtain the expression levels of *HSD17B10* and its protein in human organs or tissues [7]. The *HSD17B10* data for 33 types of cancer, together with related cancer and standard samples, were obtained from the TCGA. Then, the online tool TIMER2.0 was utilized to investigate *HSD17B10* expression in tumor versus adjacent normal tissues within the TCGA database samples [8]. The cancer data obtained from the TCGA and GTEx were combined with the gene expression matrix of standard human tissue samples and corresponding clinical data. According to the gene annotation information, the expression data of the *HSD17B10* gene were extracted from the processed dataset [9]. If there were transcripts corresponding to the *HSD17B10* gene in the dataset, the probe or transcript with the highest expression level could be selected, or the average of the expression levels of multiple probes or transcripts could be used to represent the expression level of *HSD17B10*. Then, these data were log2-transformed [10].

### 2.3. HSD17B10 Prognosis Analysis

The overall survival (OS) and disease-free survival (DFS) rates were determined based on the expression level of *HSD17B10* across various tumor patient types in the TCGA database. Forest plots and Kaplan–Meier curves were utilized to investigate the relationship between *HSD17B10* expression and patient outcomes, including OS, DFS, and PFI across 33 types of cancer. A univariate survival analysis was conducted to calculate the hazard ratios (HRs) along with their corresponding 95% confidence intervals [11]. Survival curves of patients with different gene expression levels were drawn to compare the differences in prognostic indicators such as OS time and DFS time between the high-expression and low-expression cohorts. Combining the expression or activity of the gene with the clinical pathological parameters of patients, which may involve comparing the effects of different treatment methods [12], and evaluating the role of an intervention measure in improving prognosis can more comprehensively assess the prognosis of patients.

### 2.4. Analysis of the Correlation Between HSD17B10 Expression and Immune Infiltration via Immune-Related Scores

This study used the Immune, Stromal, and ESTIMATE scores to explore the relationship between *HSD17B10* expression and immune infiltration [13]. The Immune score assesses immune cell infiltration by analyzing the expression of marker genes for immune cells and using a weighted calculation method, with a higher score indicating more infiltration. The Stromal score, based on the expression data of stromal cell marker genes and integrated by principal component analysis, shows that a higher score means a higher content and activity of stromal cells in tumors. The ESTIMATE score uses the ESTIMATE algorithm to screen characteristic genes from the TCGA database. Then, the score is calculated, where a higher score implies a better content of stromal and immune cells in tumor samples, and a lower score means the opposite.

### 2.5. HSD17B10 Gene Mutation Analysis

The cBioPortal database is a powerful resource [14] that enables researchers to analyze the mutation rate, mutation type, and copy number alteration of the *HSD17B10* in different cancers. By using the “Mutations” module of cBioPortal, researchers can view the mutation site information of the *HSD17B10* gene, and this information can be visualized in the 3D protein structure, which is helpful to understand the possible impact of mutations on protein function.

### 2.6. Study on the Relationship Between HSD17B10 and TMB, MSI, and MMR

Tumor mutation burden (TMB) is the total number of genetic alterations per million tumor cells and is a promising predictor for the success of cancer immunotherapy [15]. Meanwhile, microsatellite instability (MSI) arises from faulty DNA mismatch repair (MMR), which can lead to disorders related to gene duplication and the progression of tumors, subsequently impacting prognosis. Spearman correlation analysis was employed. MMR serves as a crucial genetic surveillance mechanism, identifying and correcting errors in nucleotide alignment during DNA replication, thus preserving genetic stability.

### 2.7. DNA and RNA Methylation Analysis of HSD17B10

There may be abnormal DNA methylation in tumor cells. The UALCAN database was searched to explore the *HSD17B10* and measure the level of DNA methylation in the promoter region of certain cancers, as well as identify the differences between tumor and normal tissues [16]. By analyzing the DNA methylation status in tumor cells, biomarkers for tumor diagnosis and prognosis can be found, and new treatment methods can be developed [17]. RNA methylation is a key RNA epigenetic modification that significantly influences gene expression regulation, RNA metabolism, tumorigenesis, development, prognosis, and various biological processes [18]. Developing therapeutic drugs targeting RNA methylation-related enzymes or pathways may provide new strategies for disease treatment. Spearman correlation analysis explored the correlation between *HSD17B10* and m6A, m5C, and m1A regulatory proteins with *p* < 0.05.

### 2.8. Drug Sensitivity Analysis

Drug sensitivity analysis is a crucial bioinformatics method that helps researchers understand the impact of different gene expression patterns or gene variations on drug response. This analysis usually involves various databases and tools, such as the Drug Sensitivity Genomics Database (GDSC). These databases provide a large amount of data on the sensitivity of cancer cell lines to different drugs [19]. In drug sensitivity analysis, R packages such as pRRophetic 1.28.0 or oncoPredict 1.22.0 may be used for prediction and analysis.

### 2.9. Co-Expressed Genes and Enrichment Analysis of HSD17B10

GeneMANIA is an online resource for investigating genetic interactions, roles, and pinpointing genes that are co-expressed [20]. Fifty genes co-expressed with *HSD17B10* were obtained through GeneMANIA. We used the LinkedOmics database to further validate the rich biological functions and pathways related to *HSD17B10* in bladder cancer (BLCA) [21]. The LinkedOmics database was utilized to calculate the correlation coefficients for genes co-expressed with *HSD17B10*. GSEA was used to study GO-BP and KEGG pathways of *HSD17B10* and its co-expressed genes. The R package “clusterProfiler 4.0+” was used for GO and KEGG enrichment. Finally, GSEA was used to study the pan-cancer biological functions across low and high expressions of *HSD17B10* using the R package clusterProfiler (a universal enrichment tool for interpreting omics data).

### 2.10. Processing of Single-Cell and Spatial Transcriptome Data

Finally, to evaluate the cytological characteristics of *HSD17B10* at the single-cell level, the expression data of 3138 colorectal cells were obtained from the GSE146771 dataset. The StomicsDB database was used to test and analyze the single-cell sequencing of colorectal cancer. To further explore the spatial expression of *HSD17B10*, spatial transcriptome data for glioma were sourced from the official 10xGenomics website [22]. The spatial transcriptome analysis process was used to group the data, and the annotation information of GSE146771 was used to annotate the cells in the spatial transcriptome.

## 3. Results

### 3.1. Differential Expression and Survival-Related Analysis of HSD17B10 in Multiple Cancers

**Figure 1:**
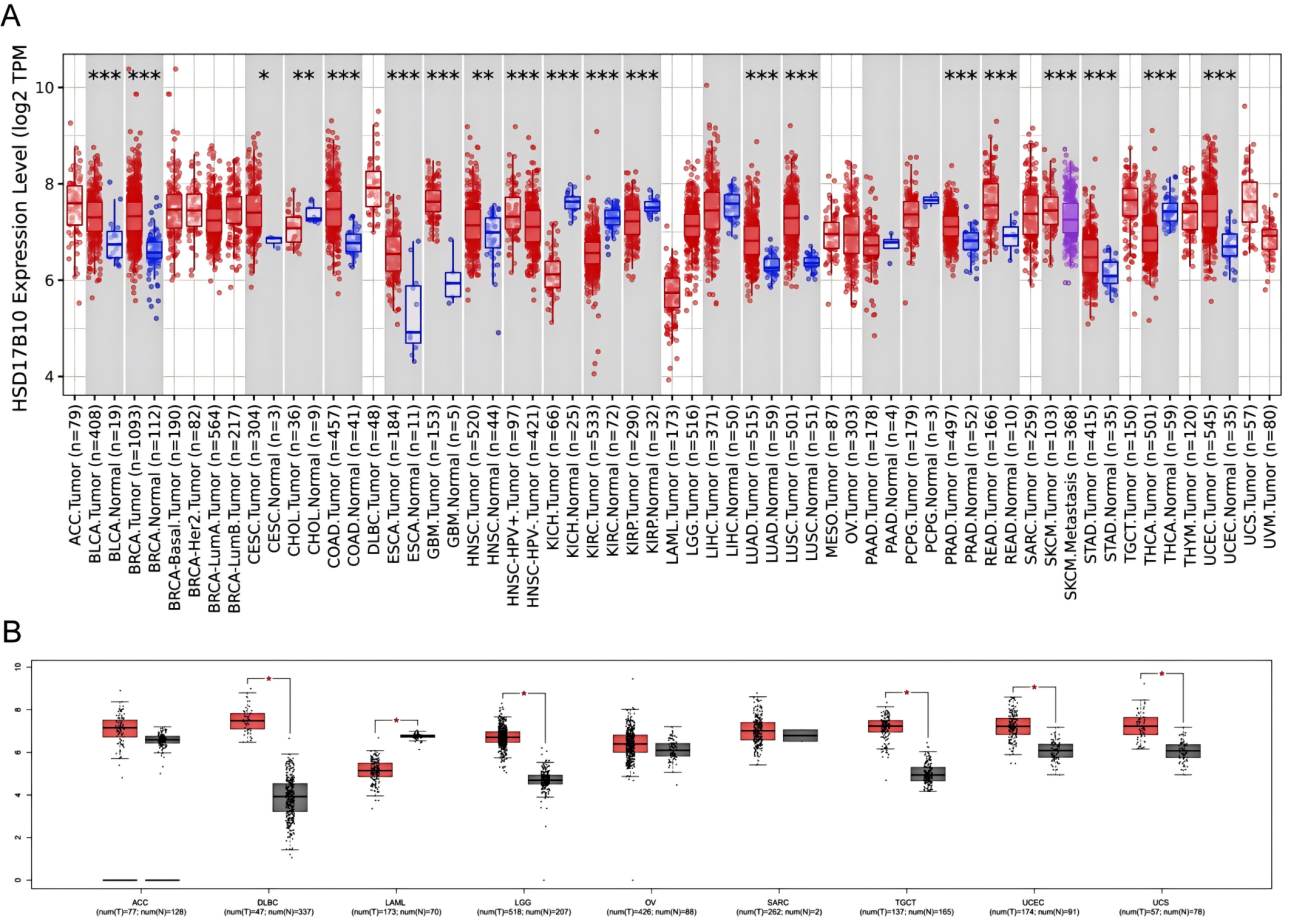
Expression differences of the *HSD17B10* gene between tumor and normal tissues. (**A**) *HSD17B10* expression levels in tumor and normal tissues from TCGA pan-cancer dataset. (**B**) *HSD17B10* expression levels in paired tumor/normal samples from TCGA. * *p* < 0.05, ** *p* < 0.01, *** *p* < 0.001.

The TIMER2 database examined *HSD17B10* expression in various cancerous and normal tissues. The levels of *HSD17B10* were found to be increased in 14 types of cancers, such as GBM, LGG, CESC, LUAD, COAD, BRCA, ESCA, STES, STAD, PRAD, UCEC, LUSC, READ, and BLCA. The expression of *HSD17B10* was downregulated in five cancers, including KIRP, KIRC, KICH, THCA, and CHOL. Considering that some cancers lacked normal tissue data in the TIMER2, we further integrated the TCGA and GTEx databases and used the GEPIA database to conduct a supplementary analysis [17]. As presented in Figure 1B, the expression of *HSD17B10* was increased in DLBCL, LGG, TGCT, THYM, UCEC, and UCS, while it was decreased in LAML.

**Figure 2:**
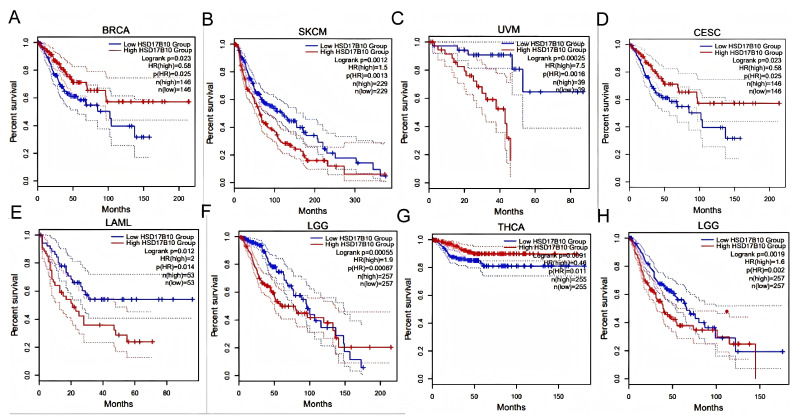
Survival outcomes of cancer patients based on high and low *HSD17B10* expression in the pan-cancer dataset using the GEPIA2 tool. (**A**–**F**) Overall survival (OS) analysis of *HSD17B10* gene expression levels in the TCGA pan-cancer dataset. (**G**,**H**) Disease-specific survival (DSS) analysis of *HSD17B10* gene expression levels in the TCGA pan-cancer dataset.

Patients were grouped by *HSD17B10* expression levels (high or low). Survival outcomes across various tumors were analyzed to assess their prognostic value.as shown in Figure 2A–F, GEPIA2 database analysis showed that in BRCA, CESC, LAML, LGG, SKCM, and UVM, high *HSD17B10* expression usually meant worse overall survival (OS, all *p* < 0.05). However, in BRCA, some results indicated that high expression was related to better OS.as shown in Figure 2G,H, In LAML, LGG, and THCA, high *HSD17B10* expression was associated with poorer disease-free survival (DFS, *p* < 0.05), yet missing data affected the presentation of LAML results.

### 3.2. Prognostic Analysis of HSD17B10 Expression

**Figure 3:**
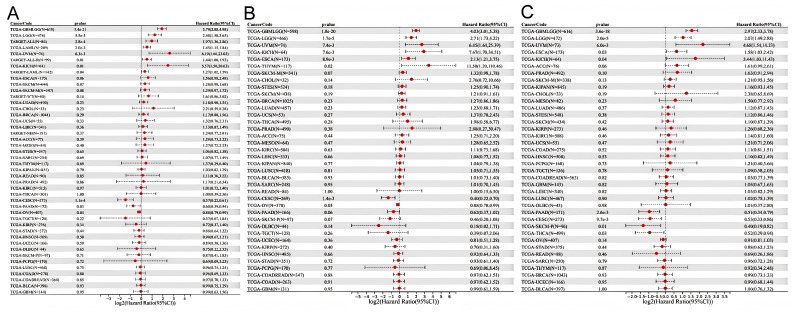
Univariate regression of OS (**A**), DSS (**B**), and PFI (**C**) in cancer.

We constructed a Cox proportional hazards regression model. OS, DSS, and PFI were selected to study the prognostic value of *HSD17B10* in pan-cancer. The Log-rank test was employed for statistical analysis to determine prognostic significance. It was observed from Figure 3A that high expression of *HSD17B10* was associated with an unfavorable OS in GBMLGG, LGG, UVM, ESCA, and KICH. However, in CESC, THCA, PAAD, and SKCM-P, low expression of *HSD17B10* was linked to a negative outcome. For DSS, high expression of *HSD17B10* was linked to a negative outcome in GBMLGG, LGG, ESCA, THYM, UVM, and KICH. In contrast, low expression of *HSD17B10* was linked to a negative outcome in CESC and OV (Figure 3B). Regarding PFI, high expression of *HSD17B10* was associated with a poor prognosis in GBMLGG, LGG, ESCA, UVM, and KICH. Conversely, low expression of *HSD17B10* was associated with a poor prognosis in CESC, THCA, PAAD, and SKCM-P (Figure 3C). In summary, in GBMLGG, LGG, ESCA, UVM, and KICH, high expression of *HSD17B10* significantly shortened OS, DSS, and PFI. In CESC, a significant negative “log2(Hazard Ratio (95%CI))” indicated that higher expression of *HSD17B10* was associated with a lower risk of poor prognosis. A significant negative score implies that high *HSD17B10* expression is related to better OS, DSS, and PFI. These results indicate that *HSD17B10* can be an independent prognostic marker for these cancers.

### 3.3. Examining the Connection Between HSD17B10 Expression and Immune Cell Infiltration in Various Cancer Datasets

**Figure 4:**
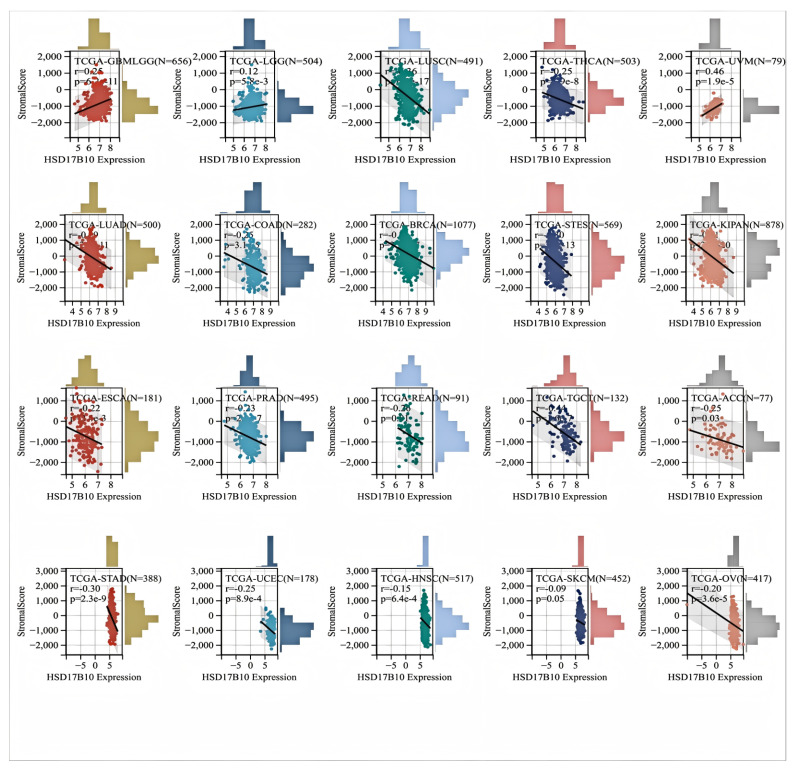
Using the ESTIMATE algorithm to perform a correlation analysis between *HSD17B10* expression and ESTIMATE scores across various cancers.

The relationship between *HSD17B10* expression and immune infiltration across various cancers was investigated by analyzing immune scores, stromal scores, and ESTIMATE scores. We can utilize the Pearson correlation coefficient to identify correlated immune–infiltration scores. In most types of cancer (Figure 4), *HSD17B10* expression showed an inverse relationship with stromal and immune scores while exhibiting a positive correlation with GBMLGG, LGG, and UVM. Eventually, the *HSD17B10* expression was notably correlated with the ESTIMATE score in 22 cancer types. Specifically, there were three significant positive correlations in GBMLGG, LGG, and UVM, suggesting that high *HSD17B10* expression might be tied to greater immune and stromal cell infiltration in these three cancers. However, in most types of cancer, its expression was inversely related to ESTIMATE. The link between *HSD17B10* and the ESTIMATE score could assist in predicting the tumor immune microenvironment and patients’ treatment responses, laying a foundation for personalized treatment.

**Figure 5:**
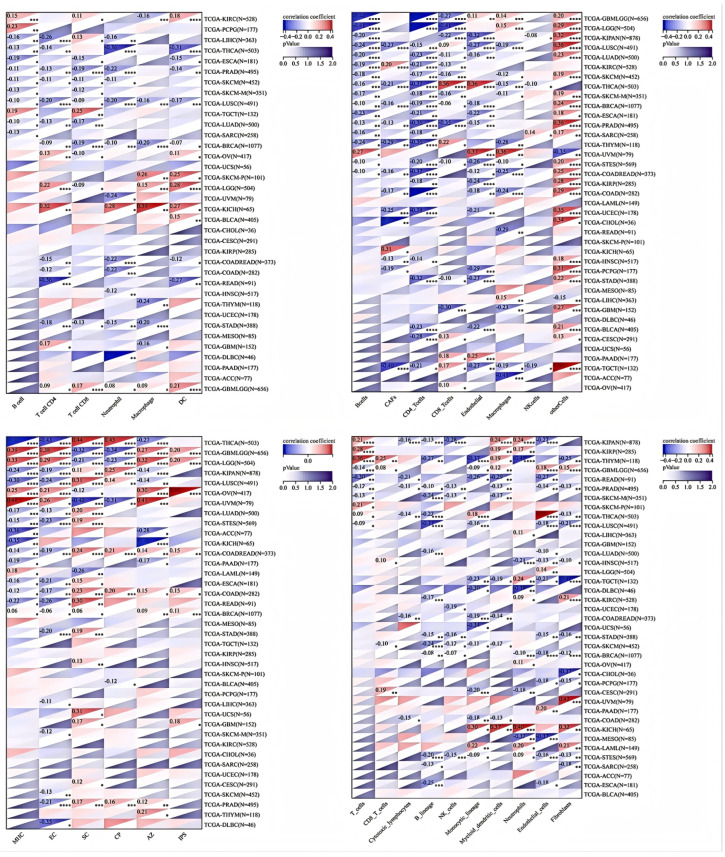
The results of TIMER, EPIC, IPS, and MCPCOUNTER analyses. * *p* < 0.05, ** *p* < 0.01, *** *p* < 0.005, **** *p* < 0.001.

This study also utilized MCPCOUNTER, IPS, TIMER, and EPIC [21,23] algorithms to examine the relationship between immune cell infiltration levels and *HSD17B10* expression in various tumor types from the TCGA database (Figure 5). The TIMER database revealed that in LUSC, *HSD17B10* expression was negatively correlated with the infiltration of B cells, T cells, neutrophils, macrophages, and DCs. Additionally, it was observed that in most tumors, the gene was negatively associated with the infiltration of immune cells. In particular, B cells, T cells, and neutrophils were negatively correlated with the expression level of *HSD17B10* in several tumors, especially PRAD, SKCM, and LUAD. The examination of the EPIC database revealed that in many tumors, especially GMBLGG and LGG, the level of *HSD17B10* expression was inversely related to the degree of T-cell infiltration in cancer. The examination of the IPS database revealed that the expression level of *HSD17B10* showed a positive correlation with IPS but an inverse relationship with the infiltration level of AZ (the expression level of a group of immune molecules closely related to the immune response in the tumor microenvironment). The results of the MCP algorithm showed that the expression level of *HSD17B10* was positively correlated with endothelial cell infiltration and negatively correlated with B cell lines, natural killer cells, and fibroblasts. These findings suggest a strong link between *HSD17B10* expression and immune infiltration, indicating that *HSD17B10* may be vital in tumor–immune interactions.

### 3.4. Correlation Between HSD17B10 Expression and ICP, TMB, MSI, and Neoantigens in Pan-Cancer Datasets

**Figure 6:**
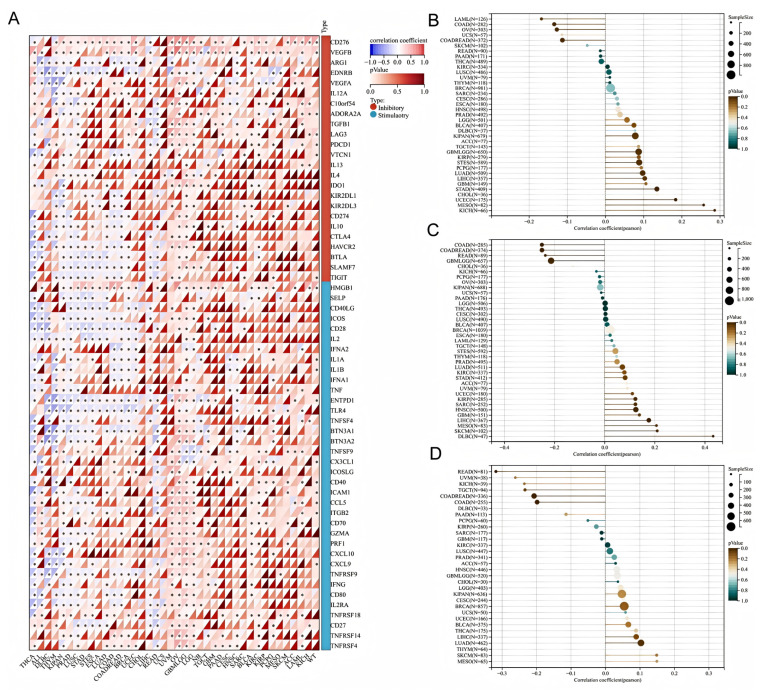
Association of *HSD17B10* with pan-cancer immune genes, TMB, MSI, and neoantigens. (**A**) The relationship between *HSD17B10* expression and pan-cancer ICP genes. (**B**) TMB, (**C**) MSI, and (**D**) neoantigens, with different colors indicating *p*-values. * *p* < 0.05.

Immune checkpoints (ICPs) are key regulators of immune cells. They regulate immune activation, maintain immune system balance, prevent autoimmune diseases, and help fight pathogens. Abnormal ICP molecule expression and function contribute to many diseases, and ICP-related genes play a significant role in tumor immune escape. As shown in Figure 6A, This study examined *HSD17B10* expression and ICP genes to understand *HSD17B10*’s role in immunotherapy. In cancers like UVM, KIPAN, KIRC, PRAD, THCA, PAAD, LIHC, and OV, *HSD17B10* expression correlated with ICP genes. In UVM, GBMLGG, LGG, KICH, BLCA, and KIRC, *HSD17B10* had positive correlations with some immune-related genes, while in THCA, READ, PRAD, LUSC, and KIPAN, it had negative correlations. Since *HSD17B10* links to immune-related genes in most tumors, it could be a potential tumor treatment target.

Previous studies showed that TMB, MSI, and neoantigens are essential for cancer immunotherapy patient response and treatment outcome prediction. We explored the correlation between *HSD17B10* levels and TMB, MSI, and neoantigen profiles in TCGA tumors. Using Pearson correlations, we found significant associations in 11 tumors. As shown in Figure 6B, For TMB, there were positive associations in GBMLGG, LUAD, STES, KIPAN, STAD, UCEC, MESO, and KICH, and negative ones in COAD, COADREAD, and OV. As shown in Figure 6C, Regarding MSI, *HSD17B10* was positively correlated in KIRP, HNSC, LIHC, SKCM, and DLBC, and negatively in GBMLGG, COAD, COADREAD, and READ (all *p* < 0.05). As shown in Figure 6D, For neoantigens, it was negatively correlated in COAD and READ patients but positively correlated in LUAD patients. These results suggest that *HSD17B10* impacts the anti-tumor immune state by regulating the tumor immune microenvironment (TME).

### 3.5. Analysis of HSD17B10 Gene Alterations

**Figure 7:**
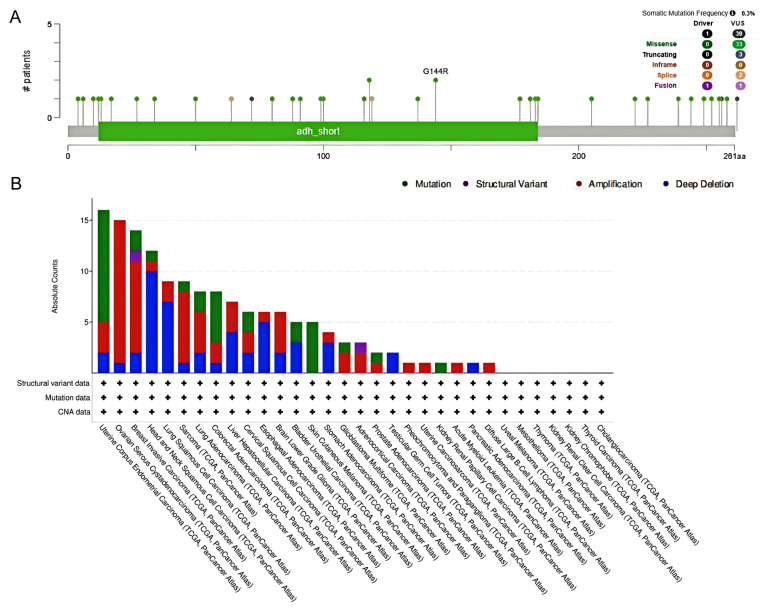
*HSD17B10* gene mutational characteristics. (**A**) Mutation frequency and types of the *HSD17B10* gene. (**B**) Mutation counts of the *HSD17B10* gene across various cancer types.

To further explore the functional mechanism of *HSD17B10*, we analyzed its genetic variations. The cBioPortal web-based application investigated the gene mutations associated with *HSD17B10* in cancer. This analysis encompassed all TCGA PanCancer Atlas studies, including 32 studies and a total of 10,967 samples. As illustrated in the figure, the frequency of somatic mutations was observed to be 0.003. A total of 39 mutation sites were identified within the range of amino acids 0 to 261, comprising 33 missense mutations, 3 truncating mutations, and 2 splicing mutations, and there were 41 SV/fusion mutations. Among them, G144R was the most common mutation site. In addition, Figure 7B shows that the mutation frequency of the *HSD17B10* was the highest in UCEC. The *HSD17B10* gene showed copy-number alterations in DLBC, UCS, LAML, and PCPG. Additionally, deep deletions were detected in the *HSD17B10* gene in TGCT and PAAD.

### 3.6. Connection Between HSD17B10 Expression and DNA and RNA Methylation

**Figure 8:**
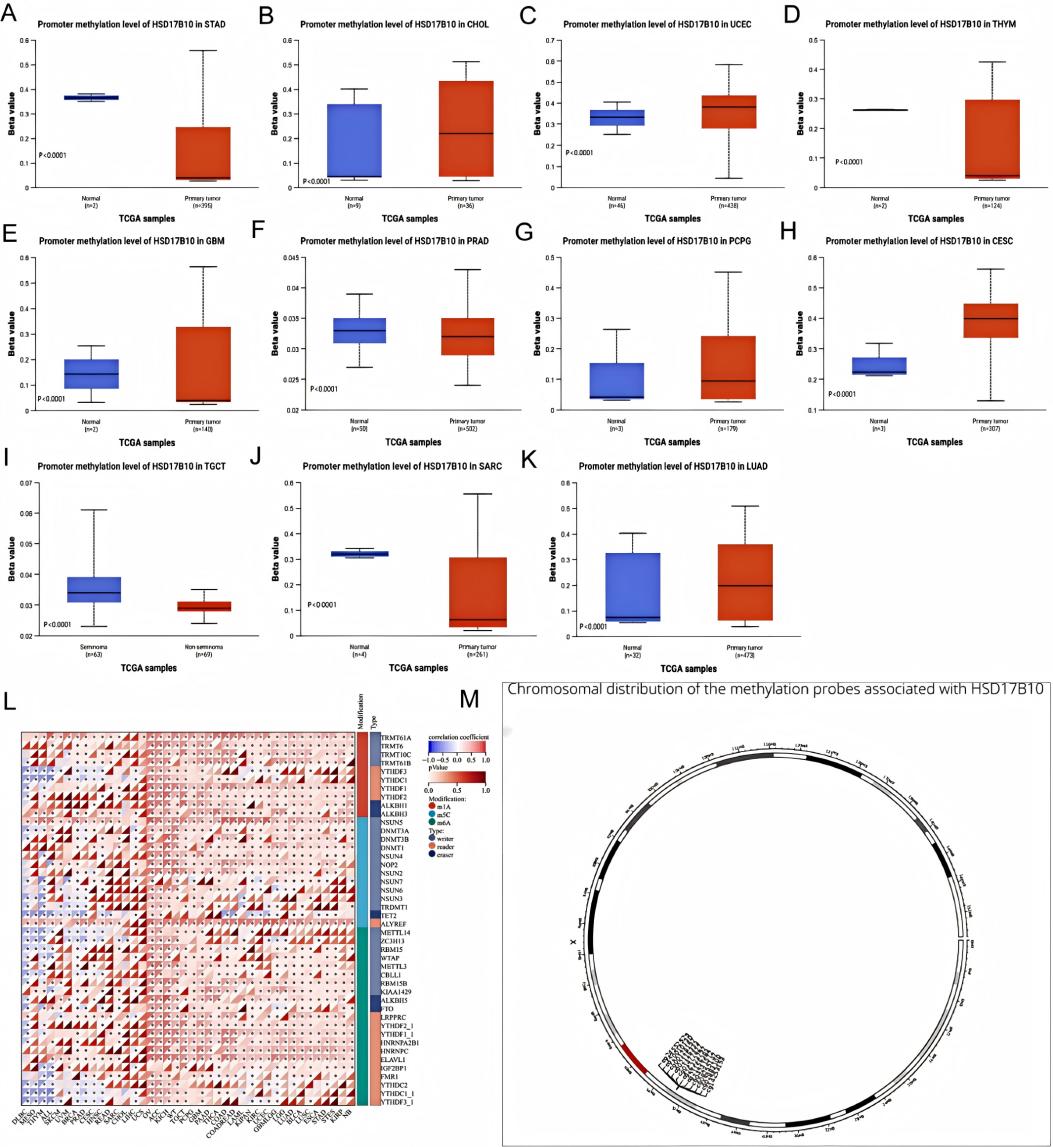
The association between *HSD17B10*, methylation status, and methyltransferases. (**A**–**K**) Promoter methylation levels of *HSD17B10* in various cancers. (**L**) Relationship between *HSD17B10* expression and m1A, m5C, and m6A, * *p* < 0.05. (**M**) The locations of methylation markers linked to *HSD17B10* on chromosomes.

This study used data from multiple databases to examine the DNA methylation level of *HSD17B10* in various tumors, comparing it between cancerous and healthy tissues, as shown in Figure 8A–K. In SARC, TGCT, STAD, THYM, GBM, and PRAD, *HSD17B10* methylation was lower than in normal tissues, perhaps explaining its high expression in these tumors. Conversely, the methylation level increased in UCEC, LUAD, PCPG, and CESC.

This study utilized the online server MethSurv to analyze the relationship between DNA methylation patterns, methylation levels at each CpG site of *HSD17B10*, and survival data. As Figure 8M shows, *HSD17B10* has 11 methylation probes: CG14253153, CG02883100, CG08255147, CG04241572, CG21156383, CG26323797, CG01477427, CG02116333, CG00128197, CG20010130, and CG24552529. According to relevant tables, high methylation of CG26323797 signaled a good prognosis in GBM, LGG, SKCM, and UCEC, but a poor one in ACC and LIHC. The high methylation of other probes also showed different prognostic implications for various cancers. For example, CG14253153 had a good prognosis in HNSC and KIRP. *HSD17B10* may affect cancer patients’ prognosis through methylation.

Furthermore, we retrieved *HSD17B10* marker genes and 44 genes related to m1A, m5C, and m6A RNA modifications in each sample. In many cancers, *HSD17B10* was positively correlated with the regulatory genes of m6A, m5C, and m1A. It may promote carcinogenesis by regulating the expression of these RNA methylation regulatory genes, suggesting a close link between *HSD17B10*’s activity or expression level and RNA methylation regulation. This can help in evaluating patient prognosis.

### 3.7. Drug Sensitivity Analysis

**Figure 9:**
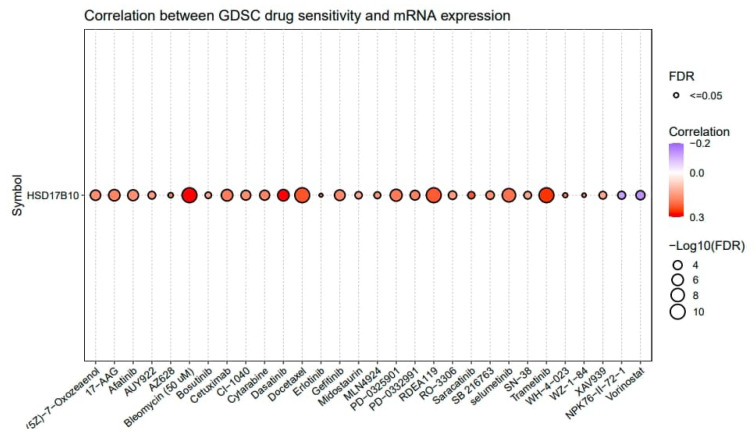
The association between *HSD17B10* expression and drug sensitivity.

This study utilized GSCALite to explore the drug sensitivity related to *HSD17B10* expression in tumors, concentrating on the sensitivity of the top 30 GDSC drugs across various cancers. If a gene is expressed more, it could make the cancer resistant to drugs, while a negative one means that increased gene expression could boost drug sensitivity. Figure 9 shows that *HSD17B10* was inversely associated with the 50% inhibitory concentration (IC50) values of NPK76-II-72-1 and Vorinostat. Additionally, the expression of *HSD17B10* was positively correlated with the IC50 of 28 drugs, including (5Z)-7-Oxozeaenol, 17-AAG, Afatinib, AUY922, AZ628, Bleomycin (50 μM), Bosutinib, Cetuximab, CI-1040, Cytarabine, and Dasatinib.

### 3.8. Functional Enrichment Analysis of HSD17B10-Related Genes

To explore *HSD17B10*’s role in tumorigenesis, we screened 30 related genes from GeneMANIA. Using GEPIA2 with TCGA tumor data, we obtained 30 genes highly linked to *HSD17B10* expression. By cross-analyzing *HSD17B10*-binding proteins in the PPI network and co-expressed genes from a Venn diagram, we identified seven key genes: NDUFB11, NDUFA13, MRPL12, TUFM, NHP2, NDUFA11, and CLPP. Integrating PPI and co-expression data, we conducted a functional set analysis for future research.

**Figure 10:**
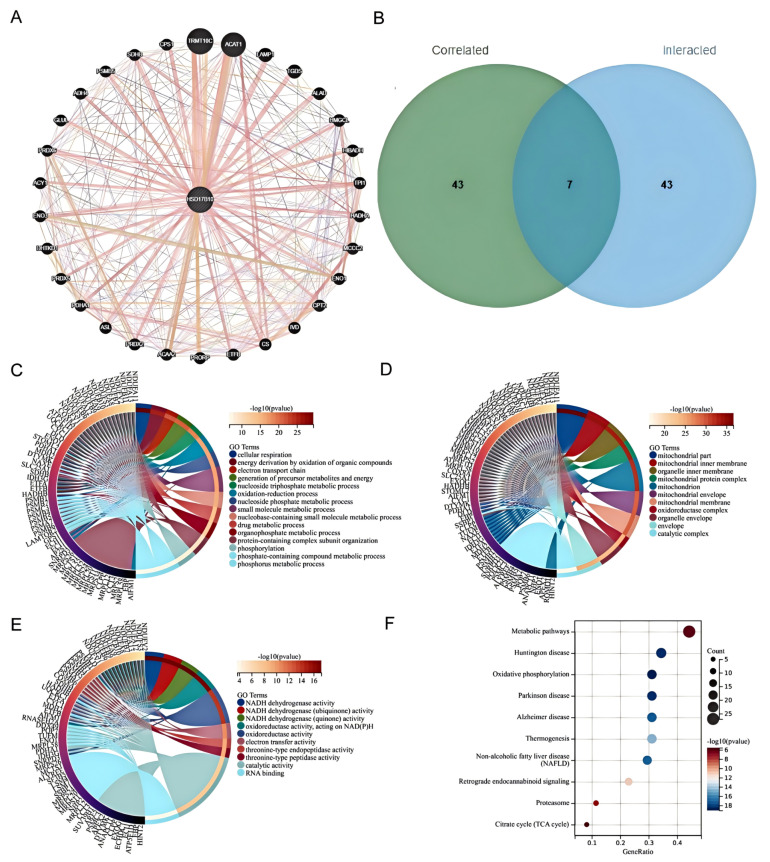
Atlas of network association and functional enrichment studies of *HSD17B10* gene in CRC. (**A**) STRING analysis identifies 50 potential *HSD17B10*-binding proteins. (**B**) Venn diagram showing the intersection between *HSD17B10*-binding genes and *HSD17B10*-related genes. (**C**–**E**) Results of GO enrichment analysis for *HSD17B10*-binding genes and *HSD17B10*-related genes. (**F**) KEGG pathway analysis for *HSD17B10*-binding genes and *HSD17B10*-related genes.

BP enrichment analysis showed that *HSD17B10* controlled the core metabolism. It was enriched in energy-related GO terms like cell respiration and redox, suggesting pathway changes (Figure 10C). Adjustments in cell respiration or energy production, along with key-node enrichment in nucleotide and small-molecule metabolism, show its role in biomolecular processes. Abnormalities can disrupt life activities and be related to diseases. CC enrichment analysis indicated that *HSD17B10* was related to mitochondrial parts and inner membranes in tumors, highlighting mitochondrial importance. Genes were involved in mitochondrial maintenance, and the presence of nuclear and organelle-related terms suggested *HSD17B10* might help with nuclear–organelle connections (Figure 10D). In MF analysis, genes around *HSD17B10* focused on oxidoreductase, with NADH dehydrogenase enrichment affecting energy and redox. Cofactor and RNA-binding enrichment showed gene-product support in the molecular network (Figure 10E). *HSD17B10* likely contributes to tumorigenesis via energy (especially mitochondrial) and redox pathways. KEGG analysis showed that *HSD17B10* was involved in disease-related metabolic pathways, and oxidative phosphorylation, with a high (Count) value, was important in energy metabolism and might link to neurodegenerative diseases (Figure 10F).

### 3.9. Validation of the HSD17B10 Co-Expression Network in BLCA

**Figure 11:**
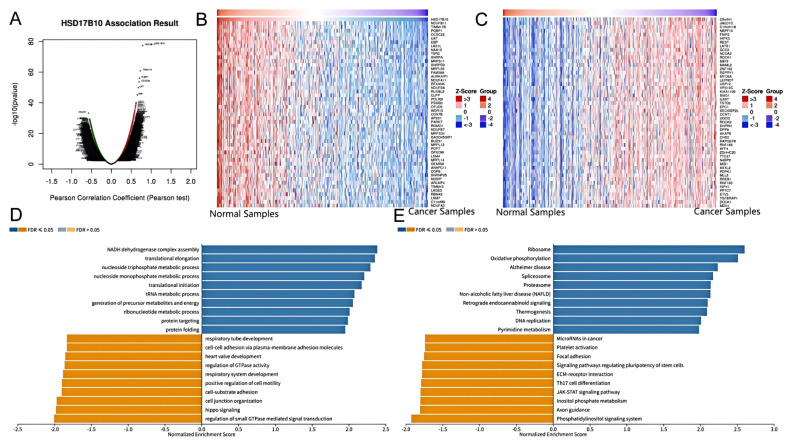
Atlas of associative studies on *HSD17B10* gene in bladder cancer (BLCA) on multi-omics analysis. (**A**) Volcano plot of *HSD17B10* co-expressed genes. (**B**) Heatmap of the top 50 genes positively correlated with *HSD17B10*. (**C**) Heatmap of the top 50 genes negatively correlated with *HSD17B10*. Red represents positively correlated genes, and blue represents negatively correlated genes. (**D**) Classification of *HSD17B10* co-expressed genes for GO analysis in bladder cancer (BLCA). (**E**) Classification of *HSD17B10* co-expressed genes for KEGG pathway analysis in *HSD17B10*.

Previous studies have confirmed *HSD17B10*’s link to tumor prognosis and immune response. This research delved into its connection with BLCA using the LinkedOmics database. We systematically analyzed related genes’ KEGG pathways and GO-BP terms using the Pearson correlation coefficient for gene ranking. Figure 11A shows the volcano plot of *HSD17B10* co-expression genes in BLCA. Most genes have non-significant correlations (black dots in the middle), while a few at the ends show significant positive or negative correlations. These significant genes might be *HSD17B10*’s potential co-expression partners in BLCA and merit further study. Figure 11B,C are heatmaps of the top 50 genes positively and negatively correlated with *HSD17B10* in BLCA. In Figure 11B (positive correlation), some genes show high expression (red areas) in specific samples, indicating a positive regulatory link with *HSD17B10*. In Figure 11C (negative correlation), some genes have low expression (blue areas), suggesting a negative regulatory link. Using the Gene Set Enrichment Analysis (GSEA) module in LinkedOmics, we identified the most enriched GO-BP terms and KEGG pathways related to *HSD17B10* co-expression genes in BLCA (Figure 11D,E). GO analysis shows high Normalized Enrichment Score (NES) values for *HSD17B10* in translational elongation, nucleoside monophosphate metabolism, cell–substrate adhesion, and Hippo signaling, potentially related to cell adhesion and signal transduction, which are crucial in cancer development. KEGG analysis reveals *HSD17B10* enrichment in ribosome, oxidative, proteasome, and phosphatidylinositol signaling systems. These results offer key insights into *HSD17B10*’s function in BLCA, guiding new treatment strategies and biomarker discovery.

### 3.10. Investigation of the Role of HSD17B10 in CRC by Single-Cell Transcriptome and Spatial Transcriptome

**Figure 12:**
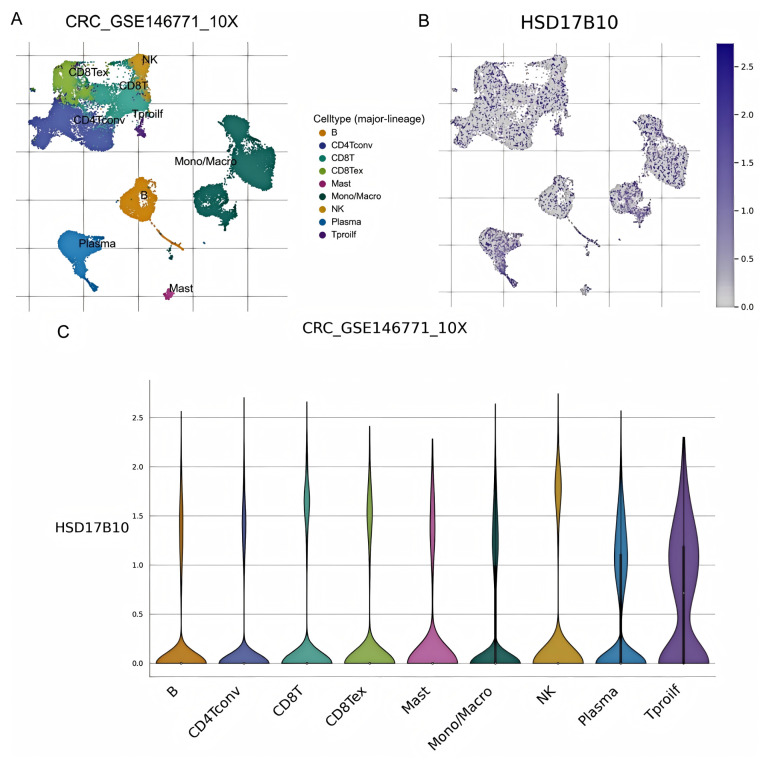
Research on colorectal cancer (CRC) and *HSD17B10* gene. (**A**) Single-cell clustering atlas of CRC. (**B**) Expression distribution of *HSD17B10* gene. (**C**) Expression differences of *HSD17B10* in various cell populations.

This study analyzed *HSD17B10*’s role at the gene expression level and explored its cell localization in colorectal cancer using the GSE146771 dataset. Single-cell transcriptome sequencing data offered a unique view of gene functions in various cell types. We examined *HSD17B10* expression in single-cell transcriptome data (Figure 12A,B). Figure 12A shows heterogeneity. Figure 12B shows the expression distribution of *HSD17B10* gene and provides specific data support for the study’s relevant conclusions about the *HSD17B10* gene. Figure 12C presents *HSD17B10* expression in different cell types via different visualizations. The violin plots in Figure 12C, with width indicating cell density at specific expression levels, shows gene expression distribution. Figure 12C indicates that *HSD17B10* expression was more concentrated in CD4 T cells, CD8 T cells, B cells, innate lymphocytes, and myeloid cells, and these cell types had similar expression patterns. Combining these figures enhanced our understanding of *HSD17B10*’s role in the tumor microenvironment. Moreover, this study classified cells by colorectal cancer stages (I–IIIc) and detailed *HSD17B10* expression changes. Analyzing spatial transcriptome information provided key clues about *HSD17B10*’s spatial location and function in tumor tissues. Combined with the cell type distribution maps, our understanding of its action mechanism in colorectal cancer deepened. Figure 12’s spatial distribution details helped clarify *HSD17B10*’s location in tumor tissues, which is crucial for studying its role in tumor development. By integrating Figure 12A with Figure 12B,C’s cell type maps, we could more comprehensively understand *HSD17B10*’s mechanism in colorectal cancer.

**Figure 13:**
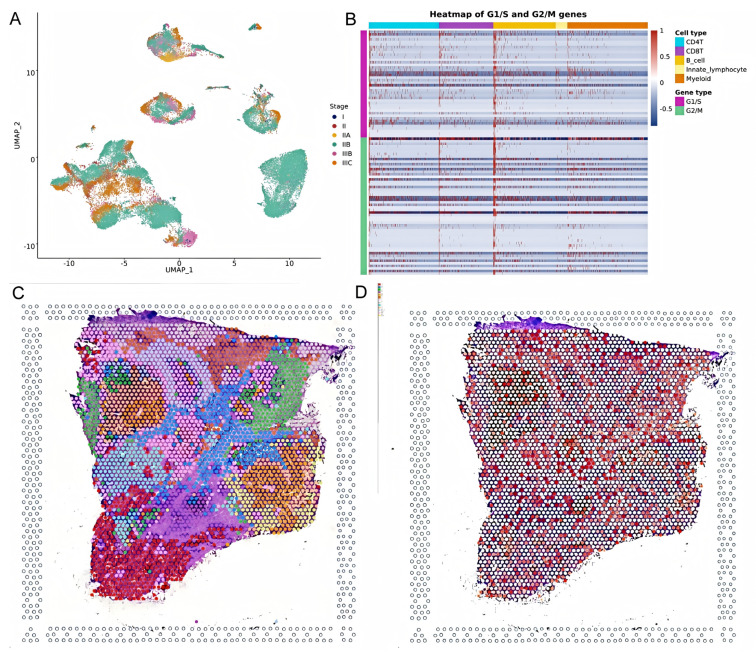
Analysis of gene expression related to colorectal cancer. (**A**) UMAP expression plot of the *HSD17B10* gene in different stages of colorectal cancer. (**B**) Heatmap expression of G1/S-phase and G2/M-phase genes in other cell types. (**C**,**D**) Spatial transcriptomics expression maps of *HSD17B10* in colorectal cancer tissue and high-resolution spatial expression maps of *HSD17B10* in colorectal cancer tissue sections.

This study shows (Figure 13A) gene expression across different cell types in the G1/S and G2/M phases. The *HSD17B10* gene may influence colorectal cancer through cell cycle regulation. Its expression can impact cell growth, oxidative stress resistance, and response to nutrient deficiency. *HSD17B10* acetylation might also regulate cell functions. As seen in Figure 10F, it is crucial for cell proliferation and survival. Its expression pattern could be tied to specific stages or subtypes of colorectal cancer, potentially offering new diagnostic and treatment biomarkers. This study also studied the spatial transcriptome of *HSD17B10* in colorectal carcinoma. Analyzing 3138 transcriptional loci, we found 10 clusters (Figure 13B). Figure 13C,D, from spatial transcriptome tech, show gene expression in tissue sections, giving key spatial info on *HSD17B10* in colorectal cancer. In summary, *HSD17B10* expression in colorectal cancer relates to cancer stage and cell types. Differences in immune cell expression may affect the tumor immune response. Spatial transcriptome data reveal its tissue location, helping us understand its role in tumor development. By studying *HSD17B10* across stages and cell types, we can better grasp its function in colorectal cancer, providing potential therapeutic targets. These analyses lay the groundwork for further *HSD17B10* research in colorectal cancer and could lead to new treatment methods.

## 4. Discussion

Previous *HSD17B10* gene studies had value but were limited. Early ones mainly explored its normal cell metabolism function. In contrast, our research is novel. We analyzed 33 cancer types in a pan-cancer study with a broader scope than before. Our study used multiple immune-related scores and algorithms to comprehensively analyze its interactions with immune infiltration in various cancers. Our findings supplement and expand existing theory, offering new perspectives on cancer development and treatment through pan-cancer expression profiling, prognostic analysis, and immune microenvironment exploration.

This study systematically analyzed the expression characteristics and clinical significance of *HSD17B10* in pan-cancer using multi-omics data from TCGA, GTEx, and other databases [23]. The results revealed that *HSD17B10* expression was upregulated in 14 cancer types, such as GBM, LGG, and LUAD, and downregulated in 5 cancer types, including KIRC and THCA. Its expression level was significantly correlated with patient survival [24]. Notably, the prognostic role of *HSD17B10* in BRCA exhibited contradictions [25], as high expression was paradoxically linked to better survival in some analyses. This may relate to tumor heterogeneity or subtype-specific regulation and requires further validation. Breast cancer encompasses multiple subtypes, including luminal A, luminal B, HER2-enriched, and triple-negative breast cancer, each with distinct molecular characteristics and clinical behaviors [26]. The contradictory results in breast cancer might stem from tumor heterogeneity. Different subtypes have diverse gene expression profiles, hormone receptor statuses, and tumor microenvironments, which could modulate the role of *HSD17B10* [27].

Analysis of the interaction between *HSD17B10* and the tumor immune microenvironment demonstrated that its expression positively correlated with ESTIMATE scores in GBM-LGG, LGG, and UVM, suggesting a potential role in promoting tumor progression through enhanced stromal and immune cell infiltration [28]. However, in most cancers like LUAD and SKCM, *HSD17B10* expression showed negative correlations with immune cell infiltration, including B cells and T cells. Additionally, *HSD17B10* expression was significantly associated with TMB, MSI, and neoantigen load [29]. For instance, it positively correlated with TMB in LUAD but negatively with MSI in COAD, implying its regulatory role in genomic instability and immune checkpoint molecules like programmed death-ligand 1 (PD-L1) that influences immunotherapy responses [30]. Epigenetic analysis revealed that reduced DNA methylation in the *HSD17B10* promoter region in cancers such as SARC and TGCT may contribute to its upregulated expression [31]. Specific methylation sites, for example, CG26323797, were significantly associated with survival in GBM and LIHC patients, highlighting the potential regulatory role of epigenetic modifications in *HSD17B10* function [32]. Furthermore, the synergistic effects of *HSD17B10* with RNA methylation regulators like m6A and m5C further support its involvement in tumor metabolic reprogramming via epigenetic networks [33].

Functional enrichment analysis indicated that *HSD17B10*-related genes were primarily enriched in oxidative phosphorylation, mitochondrial energy metabolism, and redox processes, aligning with mechanisms linking mitochondrial dysfunction to tumor progression proposed by Chatterjee et al. [34]. In BLCA, co-co-expressed genes of *HSD17B10* were implicated in Hippo signaling and ribosome functions, suggesting its role in promoting cancer progression by regulating proliferation and translational processes [35]. Single-cell and spatial transcriptomic data further revealed cell-type-specific expression patterns of *HSD17B10* in CRC, with dynamic changes observed in tumor and immune cells, providing new insights into its spatial heterogeneity.

In this study, we systematically analyzed *HSD17B10*’s expression and clinical significance across 33 cancers; however, numerous aspects remain uncharted [36]. Our research has limitations, such as dependence on public databases, inadequate experimental validation, small sample sizes for specific cancers, and unclear mechanisms of *HSD17B10*’s interactions with the epigenetic and immune microenvironments [37]. Going forward, future research should delve into the molecular signaling pathways in which *HSD17B10* is involved. Utilizing gene knockout, overexpression, and RNA interference in cell lines or animal models and comparing cellular changes under various conditions will help identify key regulatory elements [38].

## 5. Conclusions

In summary, *HSD17B10* exerts pleiotropic effects in pan-cancer by regulating mitochondrial metabolism, epigenetic modifications, and the immune microenvironment. Its expression patterns and molecular mechanisms provide a theoretical foundation for developing novel prognostic biomarkers and targeted therapies.

## Data Availability

The data of this research are publicly available.

## References

[1] Zhu M., Ma Z., Zhang X., Hang D., Yin R., Feng J., Xu L., Shen H. (2022). C-reactive protein and cancer risk: A pan-cancer study of prospective cohort and Mendelian randomization analysis. BMC Med..

[2] Sung H., Ferlay J., Siegel R.L., Laversanne M., Soerjomataram I., Jemal A., Bray F. (2021). Global cancer statistics 2020: GLOBOCAN estimates of incidence and mortality worldwide for 36 cancers in 185 countries. CA Cancer J. Clin..

[3] Hinshaw D.C., Shevde L.A. (2019). The tumor microenvironment innately modulates cancer progression. Cancer Res..

[4] Ciki K., Alavanda C., Kara M. (2024). Novel Mutation in the *HSD17B10* Gene Accompanied by Dysmorphic Findings in Female Patients. Mol. Syndr..

[5] Chatterjee A., Rodger E.J., Eccles M.R. (2018). Epigenetic drivers of tumourigenesis and cancer metastasis. Semin. Cancer Biol..

[6] Wu Q., Fu X., He X., Liu J., Li Y., Ou C. (2023). Experimental prognostic model integrating N6-methyladenosine-related programmed cell death genes in colorectal cancer. iScience.

[7] Uhlén M., Fagerberg L., Hallström B.M., Lindskog C., Oksvold P., Mardinoglu A., Sivertsson Å, Kampf C., Sjöstedt E., Asplund A. (2015). Proteomics. Tissue-based map of the human proteome. Science.

[8] Li T., Fan J., Wang B., Traugh N., Chen Q., Liu J.S., Li B., Liu X.S. (2017). TIMER: A Web Server for Comprehensive Analysis of Tumor-Infiltrating Immune Cells. Cancer Res..

[9] Yu J., Gong Y., Xu Z., Chen L., Li S., Cui Y. (2024). Prognostic and therapeutic insights into colorectal carcinoma through immunogenic cell death gene profiling. PeerJ.

[10] Wu Z., Uhl B., Gires O., Reichel C.A. (2023). A transcriptomic pan-cancer signature for survival prognostication and prediction of immunotherapy response based on endothelial senescence. J. Biomed. Sci..

[11] Zhang L., Li Y., Deng J., Liao W., Liu T., Shen F. (2024). NEK2 is a potential pan-cancer biomarker and immunotherapy target. Discov. Oncol..

[12] Ma Y.F., Chen Y., Fang D., Huang Q., Luo Z., Qin Q., Lin J., Zou C., Huang M., Meng D. (2021). The immune-related gene CD52 is a favorable biomarker for breast cancer prognosis. Gland Surg..

[13] Battaglia T.W., Mimpen I.L., Traets J.J.H., van Hoeck A., Zeverijn L.J., Geurts B.S., de Wit G.F., Noë M., Hofl I., Vos J.L. (2024). A pan-cancer analysis of the microbiome in metastatic cancer. BMC Med. Genom..

[14] Jeggo P.A., Pearl L.H., Carr A.M. (2016). DNA repair, genome stability and cancer: A historical perspective. Nat. Rev. Cancer.

[15] Liu Y., Yao Y., Yang X., Wei M., Lu B., Dong K., Lyu D., Li Y., Guan W., Huang R. (2024). Lymphocyte activation gene 3 served as a potential prognostic and immunological biomarker across various cancer types: A clinical and pan-cancer analysis. Clin. Transl. Immunol..

[16] Li Y., Xue M., Deng X., Dong L., Nguyen L.X.T., Ren L., Han L., Li C., Xue J., Zhao Z. (2023). TET2-mediated mRNA demethylation regulates leukemia stem cell homing and self-renewal. Cell Stem Cell.

[17] Liu T., Yang K., Chen J., Qi L., Zhou X., Wang P. (2023). Comprehensive Pan-Cancer Analysis of KIF18A as a Marker for Prognosis and Immunity. Biomolecules.

[18] Xie N., Shen G., Gao W., Huang Z., Huang C., Fu L. (2023). Neoantigens: Promising targets for cancer therapy. Signal Transduct. Target. Ther..

[19] Wang M., Zhu J., Ye Y., Li P., Sun W., Zhang M. (2023). N6AMT1 is a novel potential diagnostic, prognostic and immunotherapy response biomarker in pan-cancer. Bus. Strategy Environ..

[20] Wei G., Zhang H., Zhao H., Wang J., Wu N., Li L., Wu J., Zhang D. (2021). Emerging immune checkpoints in the tumor microenvironment: Implications for cancer immunotherapy. Cancer Lett..

[21] Zhang Z., Wang Z., Huang Y. (2021). Comprehensive Analyses of the Infiltrating Immune Cell Landscape and Its Clinical Significance in Hepatocellular Carcinoma. Int. J. Gen. Med..

[22] Tang Z., Li C., Kang B., Gao G., Li C., Zhang Z. (2017). GEPIA: A web server for cancer and normal gene expression profiling and interactive analyses. Nucl. Acids Res..

[23] Becht E., Giraldo N.A., Lacroix L., Buttard B., Elarouci N., Petitprez F., Selves J., Laurent-Puig P., Sautès-Fridman C., Fridman W.H. (2016). Estimating the population abundance of tissue-infiltrating immune and stromal cell populations using gene expression. Genome Biol..

[24] Pfeifer G.P. (2018). Defining driver DNA methylation changes in human cancers. Int. J. Mol. Sci..

[25] Zhang Q., Luo Y., Qian B., Cao X., Xu C., Guo K., Wan R., Jiang Y., Wang T., Mei Z. (2024). A systematic pan-cancer analysis identifies LDHA as a novel immunological, prognostic, and immunotherapy resistance predictor. Aging.

[26] Yarchoan M., Hopkins A., Jaffee E.M. (2017). Tumor mutational burden and response rate to PD-1 inhibition. N. Engl. J. Med..

[27] Darragh L.B., Oweida A.J., Karam S.D. (2018). Overcoming resistance to combination radiation-immunotherapy: A focus on contributing pathways within the tumor microenvironment. Front. Immunol..

[28] Chen Y., McAndrews K.M., Kalluri R. (2021). Clinical and therapeutic relevance of cancer-associated fibroblasts. Nat. Rev. Clin. Oncol..

[29] Ye Z., Zhong Y., Zhang Z. (2024). Pan-cancer multi-omics analysis of PTBP1 reveals it as an inflammatory, progressive and prognostic marker in glioma. Sci. Rep..

[30] Kashani B., Zandi Z., Pourbagheri-Sigaroodi A., Bashash D., Ghaffari S.H. (2021). The role of toll-like receptor 4 (TLR4) in cancer progression: A possible therapeutic target?. J. Cell. Physiol..

[31] Jia Y., Guo B., Zhang W., Wang F., Zhang Y., Zhang Q., Li E. (2023). Pan-cancer analysis of the prognostic and immunological role of GJB2: A potential target for survival and immunotherapy. Front. Oncol..

[32] Bertolin G., Jacoupy M., Traver S., Ferrando-Miguel R., Saint Georges T., Grenier K., Ardila-Osorio H., Muriel M.P., Takahashi H., Lees A.J. (2015). Parkin maintains mitochondrial levels of the protective Parkinson’s disease-related enzyme 17-*β* hydroxysteroid dehydrogenase type 10. Cell Death Differ..

[33] Lu B., Liu Y., Yao Y., Zhu D., Zhang X., Dong K., Xu X., Lv D., Zhao Z., Zhang H. (2024). Reveiling the unique role of TSPAN7 across tumors: A pan-cancer study incorporating retrospective clinical research and bioinformatic analysis. Biol. Direct..

[34] Yan B., Guo J., Deng S., Chen D., Huang M. (2023). A pan-cancer analysis of the role of USP5 in human cancers. Sci Rep..

[35] Li K., Liu J., Yang X., Tu Z., Huang K., Zhu X. (2021). Pan-cancer analysis of N4-acetylcytidine adaptor THUMPD1 as a predictor for prognosis and immunotherapy. Biosci. Rep..

[36] Guan Z., Luo L., Liu S., Guan Z., Zhang Q., Wu Z., Tao K. (2022). The role of TGR5 as an onco-immunological biomarker in tumor staging and prognosis by encompassing the tumor microenvironment. Front. Oncol..

[37] Chen G., Luo D., Zhong N., Li D., Zheng J., Liao H., Li Z., Lin X., Chen Q., Zhang C. (2022). GPC2 Is a Potential Diagnostic, Immunological, and Prognostic Biomarker in Pan-Cancer. Front. Immunol..

[38] Zhang W., Chen L., Liu J., Chen B. (2024). Inhibition of autophagy-related protein 7 enhances anti-tumor immune response and improves efficacy of immune checkpoint blockade in microsatellite instability colorectal cancer. J. Exp. Clin. Cancer Res..

